# Potential of Novel *Methyl Jasmonate* Analogs as Anticancer Agents to Metabolically Target HK-2 Activity in Glioblastoma Cells

**DOI:** 10.3389/fphar.2022.828400

**Published:** 2022-05-23

**Authors:** Damla Uludağ, Sadık Bay, Bilgesu Onur Sucu, Özgecan Şavluğ İpek, Thomas Mohr, Mustafa Güzel, Nihal Karakaş

**Affiliations:** ^1^ Cancer Research Center, Research Institute for Health Sciences and Technologies (SABITA), İstanbul Medipol University, İstanbul, Turkey; ^2^ Regenerative and Restorative Medicine Research Center (REMER), Research Institute for Health Sciences and Technologies (SABITA), İstanbul Medipol University, İstanbul, Turkey; ^3^ Center of Drug Discovery and Development, Research Institute for Health Sciences and Technologies (SABITA), İstanbul Medipol University, İstanbul, Turkey; ^4^ Medical Laboratory Techniques, Vocational School of Health Services, Istanbul Medipol University, Istanbul, Turkey; ^5^ Center for Cancer Research, Medical University of Vienna, Vienna, Austria; ^6^ ScienceConsult—DI Thomas Mohr KG, Vienna, Austria; ^7^ Department of Medical Pharmacology, International School of Medicine, Istanbul Medipol University, Istanbul, Turkey; ^8^ Department of Medical Biology, School of Medicine, İstanbul Medipol University, İstanbul, Turkey

**Keywords:** hexokinase-II, methyl jasmonate, 2DG, glioblastoma, apoptosis, autophagy, necrosis

## Abstract

Change in the energy metabolism of cancer cells, which display significant differences compared to normal cells, is a rising phenomenon in developing new therapeutic approaches against cancers. One of the metabolic enzymes, hexokinase-II (HK-II) is involved in glycolysis, and inhibiting the HK-II activity may be a potential metabolic target for cancer therapy as most of the drugs in clinical use act on DNA damage. Methyl jasmonate (MJ) is one of the compounds blocking HK-II activity in cancer cells. In a previous study, we showed that the novel MJ analogs inhibit HK-II activity through VDAC detachment from the mitochondria. In this study, to evaluate the potential of targeting HK-2 activity, through patient cohort analysis, we first determined HK-2 expression levels and prognostic significance in highly lethal glioblastoma (GBM) brain tumor. We then examined the *in vitro* therapeutic effects of the novel analogs in the GBM cells. Here, we report that, among all, compound-10 (C-10) showed significant *in vitro* therapeutic efficacy as compared to MJ which is in use for preclinical and clinical studies. Afterward, we analyzed cell death triggered by C-10 in two different GBM cell lines. We found that C-10 treatment increased the apoptotic/necrotic cells and autophagy in GBM cells. The newly developed analog, C-10, was found to be lethal against GBM by the activation of cell death authorities, mostly in a necrotic and autophagic fashion at the early stages of the treatment. Considering that possibly decreased intracellular ATP levels by C-10 mediated inhibition of HK-2 activity and disabled VDAC interaction, a more detailed analysis of HK-2 inhibition–mediated cell death can provide a deep understanding of the mechanism of action on the oncosis/necroptosis axis. These findings provide an option to design clinically relevant and effective novel HK-II inhibitors and suggest novel MJ analogs to further study them as potential anticancer agents against GBM.

## Graphical Abstract

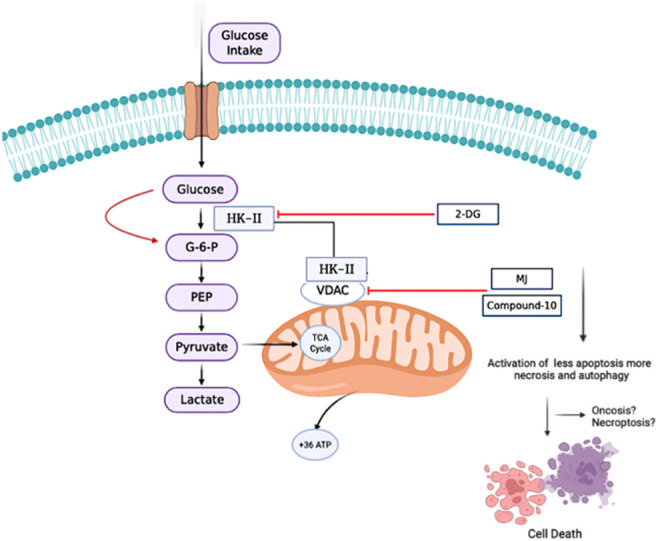

## 1 Introduction

Glioblastoma (GBM) is the most common, aggressive, and malignant brain tumor in adults. The mean survival time after diagnosis is 12–15 months (Ahmed et al., 2014; Kudulaiti et al., 2021). The main treatment options for glioblastoma include adjuvant radiotherapy and chemotherapy following surgical resection. These therapies used to target glioblastoma, being resistant to many treatments, are insufficient and associated with clinical outcomes in the poor prognosis of the disease (Fabian et al., 2019; Lukas et al., 2019). Some of the main problems in the poor prognosis are: the heterogenous structure of cancer cell populations and the genetic background and metabolic processes displaying dynamic differences (Seyfried et al., 2011; Li et al., 2016; Qazi et al., 2017; Quinones and Le, 2018; and Shergalis et al., 2018). However, targeting metabolic processes such as glycolysis is considered to be a highly discriminative and effective approach in the development of new therapeutic strategies.

Reprogramed energy metabolism is a key feature of many rapidly developing cancers, and targeting increased glycolysis in cancer cells is a new and promising therapeutic invention. Cancer cells develop various mechanisms by altering their metabolic behavior to survive in an unfavorable microenvironment (Deberardinis et al., 2008; Pinheiro et al., 2015; Xing et al., 2015; Yoshida, 2015; Rossi et al., 2017; and Chen et al., 2020). The Warburg effect, a classic metabolic adaptation of these cells, supports a metabolic shift due to anerobic glycolysis, even in the presence of sufficient oxygen. Under the Warburg effect, cancer cells produce energy with high glucose consumption and then perform lactate fermentation in the cytoplasm. This effect is thought to support proliferation, increased invasive ability, and apoptosis resistance in cancer cells (Chen et al., 2007; Hsu and Sabatini, 2008; Bensinger and Christofk, 2012; and Duraj et al., 2021). However, the enzyme hexokinase-II (HK-II) plays an important role in the development of the Warburg phenotype. HK-II catalyzes the phosphorylation of glucose to glucose-6-phosphate (G-6-P) by transferring the phosphate from ATP, the first step of the glycolytic pathway (Mathupala et al., 2009; Lis et al., 2016; and Liu et al., 2017). Therefore, HK-II has a critical role in the progression of cancer and its overexpression is associated with poor prognosis in many types of cancers, including GBMs (Chen et al., 2014; Anderson et al., 2017; and Wu et al., 2017). Overexpression of HK-II in various cancer cells has been shown to play a key role in regulating metabolic pathways.

Currently, four clinical and preclinical compounds have been reported as HK-II inhibitors, namely, methyl jasmonate (MJ) (Cesari et al., 2014), 3-bromopyruvate (3-BP), 2-deoxyglucose (2-DG), and lonidamine. MJ selectively induces autophagy, apoptosis, and non-apoptotic cell death in cancer cells (Zhang et al., 2016). Previous studies have shown that MJ suppresses proliferation and triggers cell death in a variety of human and mouse cancer cell lines, including breast, prostate, melanoma, lymphoblastic leukemia, and lymphoma cells (Zhang et al., 2015). These results have triggered many research interests as a class of multifaceted bioactive molecules to jasmonates. In a previous study, we demonstrated that new MJ analogs inhibited HK-II activity by VDAC separation from the mitochondria (Sucu et al., 2019). In another study, mitochondrial damage by certain inducers and JNK activation upon ROS production was also found in relation with VDAC opening. Downstream of this signaling is also linked to different cell death mechanisms such as necroptosis (Heslop et al., 2020). Cell death caused by the depletion of intracellular ATP levels was also reported in oncosis. In this type of cell death, the cells cannot be programed to death (apoptosis) due to low ATP, and accordingly, they show increased autophagy and necrosis.

In the current study, we focused on the most lethal cancer GBM linked to HK-II overexpression with poor prognosis and examined the therapeutic effects of these novel MJ derivatives in glioblastoma cancer cells *in vitro*. In short, our findings showed that C-10 is the most potential compound against GBM cells among newly synthesized MJ analogs. According to viability assays, as compared to MJ, C-10 induces three to four times more superior cell death in the GBM cells tested. Overall, C-10 orchestrates cell death mechanisms in GBM cells, and further *in vitro* investigations and *in vivo* tumor burden imaging of potential MJ analogs in a clinically relevant GBM model may provide more information to consider MJ derivative compounds as a GBM-targeting therapeutic agent in a clinical aspect.

## 2 Materials and Methods

### 2.1 Bioinformatics

Raw data (E-GEOD-16011 and E-GEOD-4271) were downloaded from the ArrayAxpress database using the R package ArrayExpress (Kauffmann et al., 2009). The data were combined and controlled for potential batch effects using the sva package (Leek et al., 2012). “Study” was considered a known batch effect.

The data were preprocessed using the R package “arrayQualityMetrics” (Kauffmann et al., 2009). Arrays indicated as outliers in any category (distance between arrays, boxplots, relative log expression, normalized unscaled standard error, MA plots, and spatial distribution) were excluded from further analysis. Subsequently, the arrays were RMA-normalized using the R-package “affy” (Irizarry et al., 2003). The resulting expression sets were subjected to a second QC analysis, as described previously. Finally, the probes matching multiple genes, antisense RNAs, pseudogenes, or uncharacterized loci were removed prior to further analysis.

#### 2.1.1 Threshold Setting and Survival Analysis

Thresholds were set by maximizing the log-rank statistics using the survminer package of R (Kassambara et al., 2017). Briefly, this method sets the threshold for gene expression in a way that the log-rank statistics is maximized, leading to a maximal difference between Kaplan–Meyer curves.

#### 2.1.2 Determination of Differentially Expressed Genes and Pathways

Differentially expressed genes were determined using the R package LIMMA (Ritchie et al., 2015). In addition, differentially expressed pathways were analyzed using the gene set variation analysis with Wikipathways as input (Hanzelmann et al., 2013).

### 2.2 Cell Viability Assay

Glioblastoma cancer cell lines U87-MG (ATCC # HTB-14) and LN229 (ATCC # CRL-2611) were obtained from ATCC (United States) for use in cell culture experiments. To determine GBM cancer cell populations that can be targeted by C-10, these cells were preferred as they respond differently to temozolomide (TMZ), which is the standard chemotherapeutic agent to treat GBM patients (Lee, 2016; Shaw et al., 2021). The cells were cultured with 10% FBS (Gibco), high-glucose DMEM (Gibco), and 1% penicillin/streptomycin (Gibco). The cells were incubated at 37°C in a 5% CO_2_ incubator. After achieving confluence, 5 × 10^3^ cells/well were seeded in 96-well black plates and treated with the novel molecules synthesized for 24 h at four different ranges (1–10 mM), and each concentration was prepared as triplicates. DMSO was used as control. Three independent experiments were performed. Cell viability analysis was performed according to the instructions of the Cell Titer-Glo (Promega) manufacturer. The luminescence signal was read on the SpectraMax i3x Multi-Mode Detection Platform (Moleculer Devices, United States) to determine the percentage of viable cells. IC_50_ values were determined by Graphpad Prism 7.

### 2.3 Total Protein Extraction

U87-MG and LN229 cells were seeded in a six-well plate at a density of 2 × 10^5^. According to the results obtained by the cell viability analysis, these cells were treated with C-10, MJ, and 2-DG molecules at a dose of 5 mM for 24 h. After 24 h, the medium was removed and centrifuged to collect dead cells with no surface adhesion. The cells were obtained from the wells in RIPA (Thermo Fischer) buffer with protease inhibitor (Roche, cOmpleteTM, Mini, EDTA-free Protease Inhibitor Cocktail). The cells were collected as lysate and incubated at +4°C on the shaker for 20 min. Next, the lysate was centrifuged, and the supernatant was collected. Then, the pellet was lysed in RIPA buffer. The amount of protein for each sample was determined using the nanodrop (Thermo Fisher) protein concentration calculator.

### 2.4 Western Blot

U87-MG and LN229 cells were treated with C-10, MJ, and 2-DG molecules at 5 mM for 24 h to determine the expression levels for apoptotic and necrotic markers. The cells were incubated at +4°C on the shaker for 30 min using RIPA lysis buffer with a protease inhibitor. The pellets of all samples were then collected. The total protein samples were then treated with 2X Laemli buffer (BioRad). All protein samples were loaded in a 4–15% gel at an equal concentration (25 µg) and run in an electrophoresis tank. The proteins were then transferred to polyvinylidene difluoride (PVDF) membranes using the Trans-Blot TurboTransfer System (BioRad). Following transfer, the membranes were shaken in 5% non-fat milk dissolved in 1X TBST (137 mM NaCl, 20 mM Tris, 0.1% Tween-20) RT for 1 h. The membranes were incubated with primary antibodies overnight at +4°C (anti-LC3 (Sigma #L8918) (1: 1,000). The next day, the membranes were washed three times with 1X TBST for 5 min and then with an HRP-conjugated secondary antibody (anti-rabbit CST # 7074S) (1: 2000) 5% non-fat milk dissolved in 1X TBST (137 mM NaCl, 20 mM Tris, 0.1% Tween-20) incubated at room temperature for 1 h. The protein bands were detected by ChemiDoc MP System (Bio-Rad) using the ECL HRP substrate (Biorad). The protein levels were analyzed using ImageJ, and the *β*-actin bands were used for normalization.

### 2.5 Annexin V–PI Detection

For the detection of apoptotic–necrotic cell death in U87-MG and LN229, the cells were seeded at a density of 2 × 10^5^ in six-well plates. After cell viability analysis, the cells were treated for 24 h with a 5 mM C-10 and control groups treated with DMSO accordingly. After 24 h, the cells were removed with 0.25% trypsin (Gibco) and centrifuged in the presence of 1X PBS (Gibco). The resulting pellet was re-suspended with 1X Annexin V binding buffer, and the annexin V-FITC conjugate was added and incubated on ice for 10 min. The cells were incubated with propidium iodide (PI, 1 mg/ml, Sigma) for 10 min. Cell death was analyzed in BD Biosciences-US FACS by adding 1X Annexin V binding buffer.

### 2.6 Immunofluorescence Staining

Immunofluorescence staining was performed to determine the presence of LC3 in U87-MG and LN229 cells in the last step of autophagy. The cells to be treated at a dose of 5 mM and DMSO control groups were seeded at a density of 2 × 10^5^ cells into a 35-mm petri dish. After 30 min of treatment with C-10, all cell groups were fixed with 4% PFA. After blocking the cells, primary antibody addition (LC3 1:100, CST) was performed and incubated overnight at +4°C. A secondary antibody (Alexa fluor 488, 1:200) was added to all groups and incubated at room temperature for 2 h. Then, DAPI nuclear staining, LSM 800 (Zeiss Airyscan), was performed. After quantitative measurements of the pixels were obtained with Zeiss software, each data (n) were analyzed in the SPSS program using Student’s t-test (*n* = 10).

### 2.7 Acridine Orange Staining

There are several ways to study autophagy in cells. Among these, acridine orange (AO) staining is one of the acceptable tools to detect autophagic cells through the imaging of acidic compartments (Thome et al., 2016; SenthilKumar et al., 2019). Therefore, we detected autophagy in the U87-MG after treatment with the C-10 by AO staining. First, the cells were seeded into a 35-mm glass petri dish with a density of 2 × 10^5^. After 24 h, the medium was replaced with 5 mM C-10 and subsequently, acridine- orange with a final concentration of 1 μg/ml was added. The U87-MG was real-time imaged in a confocal microscope (Zeiss LSM 880 Confocal Laser Scanning Microscope) for 24 h. The excitation laser for green fluorescence was 473 nm and for red fluorescence was 559 nm. The emission filters were 520 and 572 nm. Acridine-Orange–stained cells were imaged with the same parameters. Quantitative measures of pixels were obtained with ImageJ software (Thome et al., 2016). A statistical analysis of the results was made using one-way ANOVA in SPSS software.

### 2.8 Statistics

Student’s t-test was applied for two group comparisons except for the analysis of acridine orange staining. For the comparison of fluorescence intensities using acridine orange, one-way ANOVA test was used. All statistical analyses were performed using SPSS (IBM) software.

## 3 Results

### 3.1 Glioma Cohort Data Indicates That HK-II Overexpression is Linked to Poor Glioma Prognosis

**FIGURE 1:**
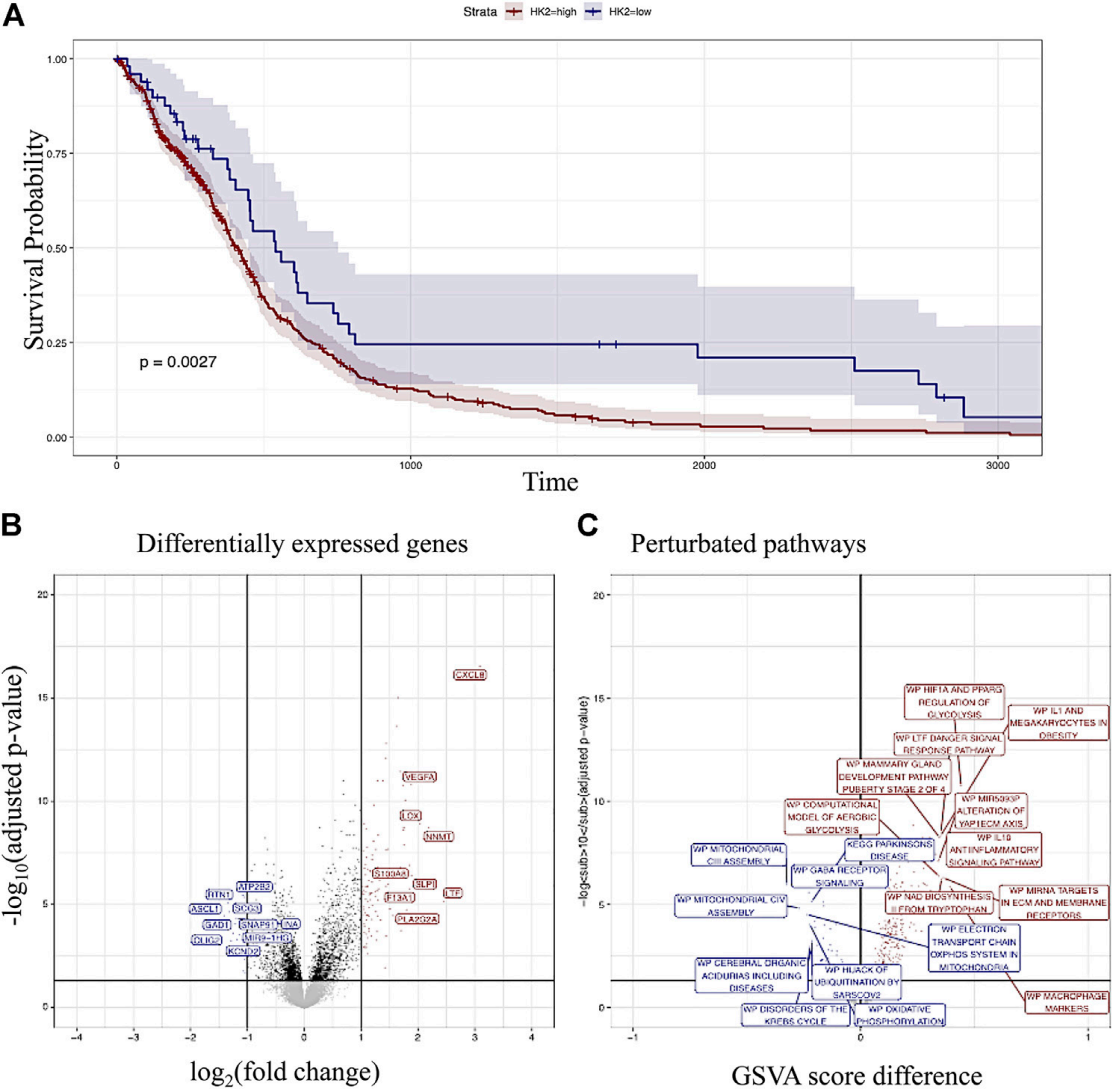
High expressors of HK-II display poor prognosis according to the glioma cohort analysis. Kaplan–Meyer analysis of HK-II high versus low expressors **(A)**. Differentially expressed genes in HK-II high versus low expressors **(B)**. Differentially regulated pathways in HK_II high versus low expressors **(C)**.

Comparison of survival between HK-II low and high expression indicated increased survival in the HK-II low expressors (Figure 1A). Analysis of differentially expressed genes revealed several differentially expressed genes in the HK-II high expressors associated with tumor progression (for example, VEGFA, CXCL8, and S100A being up-regulated (Figure 1B)). Several pathways associated with tumor progression have been found to be upregulated in HK-II high expressors (for example, the HIF1A regulation of glycolysis or IL1 and megakaryocytes in obesity, (Figure 1C)).

### 3.2 Novel MJ Analog *C-10* is More Effective in GBM Cell Death than MJ

**FIGURE 2:**
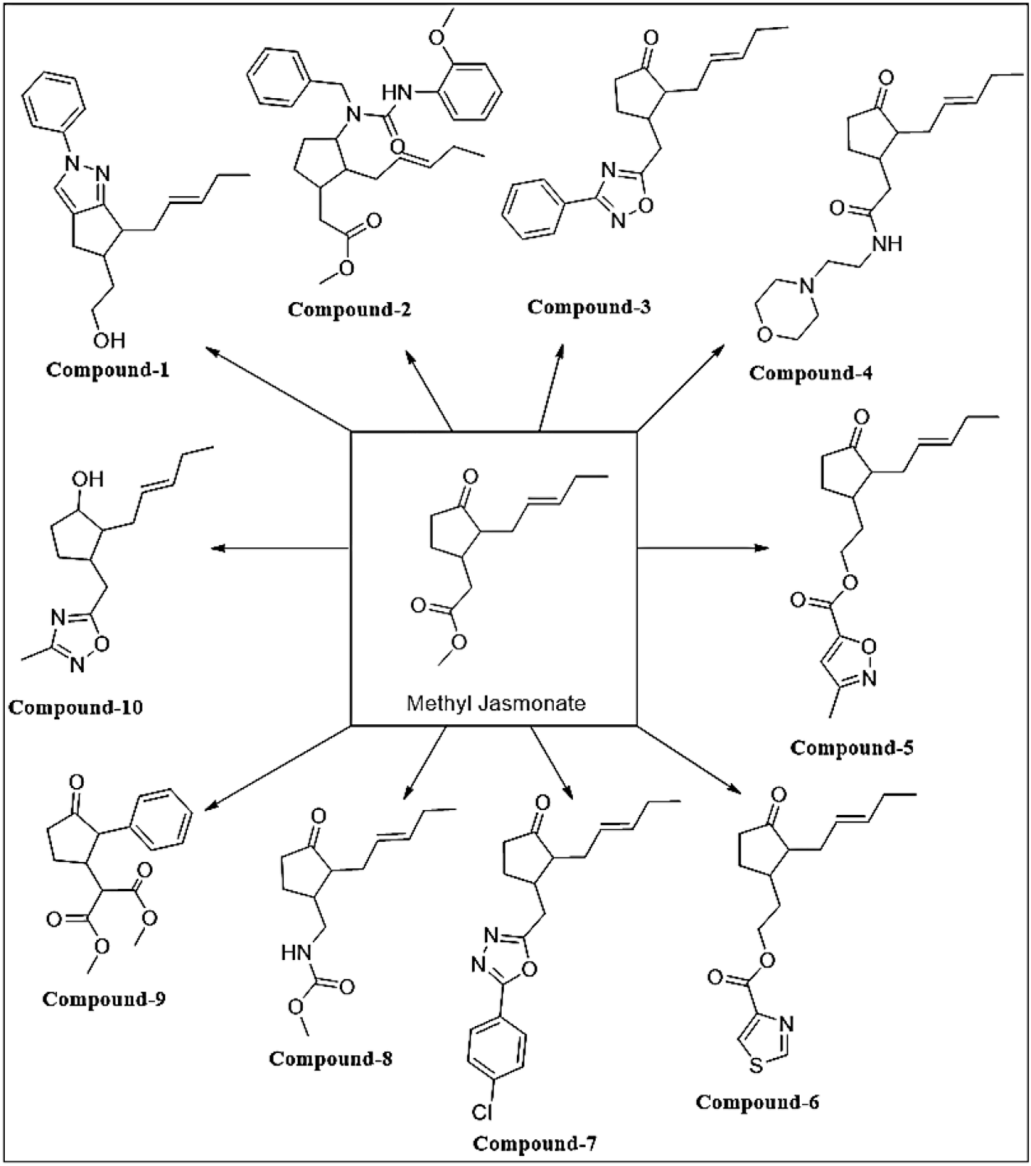
Designed, synthesized and screened novel Methyl Jasmonate analogs as HK-2 inhibitors.

**FIGURE 3:**
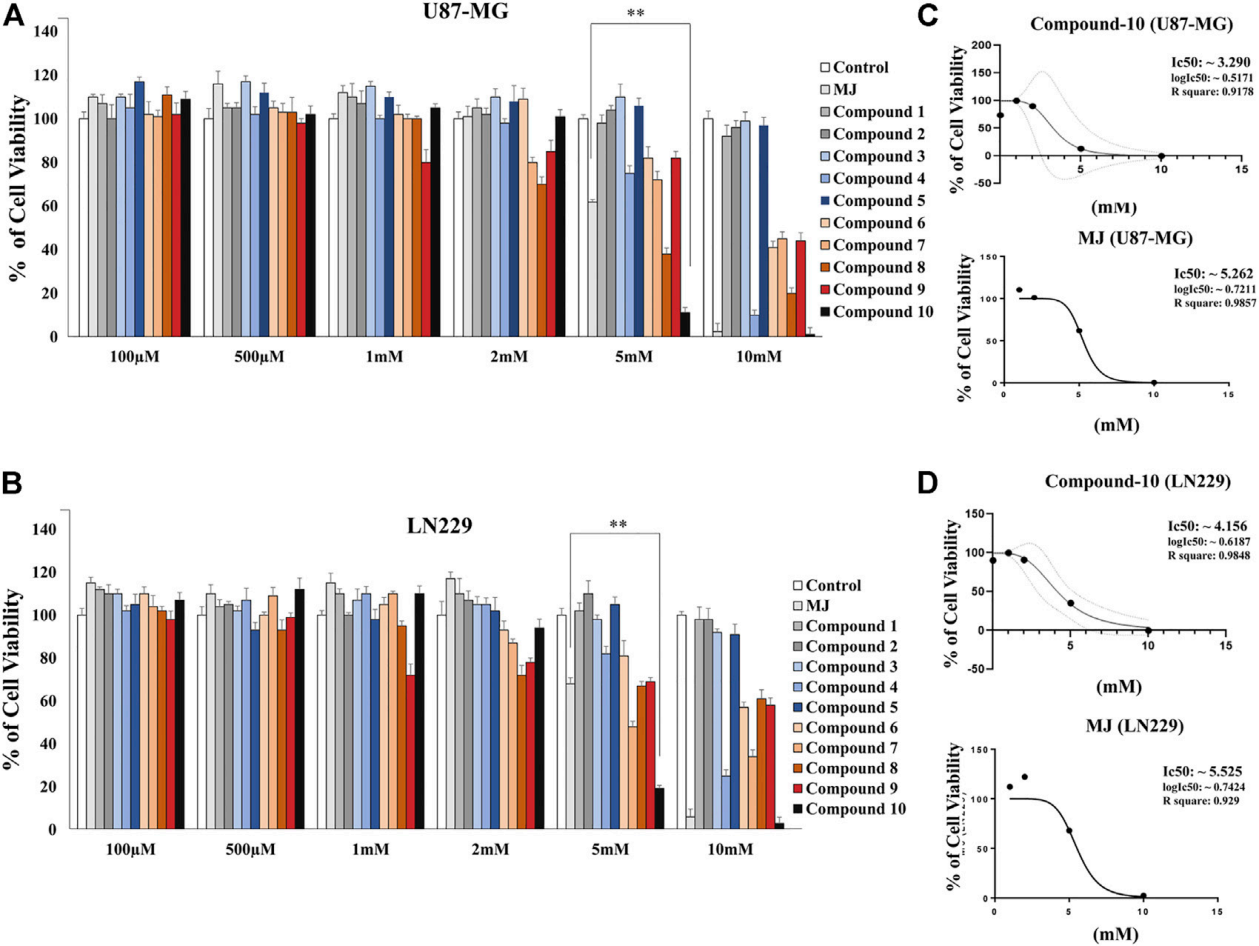
Response to the treatment with a panel of novel MJ analogs in GBM cells. Plot showing the percent viability of U-87 cells **(A)** and LN229 cells **(B)** treated with 10 different compounds at increasing doses (100 μM-10 mM) for 24 h. Data are expressed as mean ± SEM. Differences are considered significant at *p < 0.01 and **p < 0.001. Determination of the IC_50_ values of the MJ analog C-10, which is the most effective among the 10 different compounds compared to MJ, known as an HK-II inhibitor, in U-87 **(C)** and LN229 cells **(D)**.

Determining the most effective MJ analog on GBM cells, we first measured cell viability in GBM cells upon treatment with 10 different MJ-derived analogs that we previously designed (Sucu et al., 2019). Some modifications were made to the chemical structures of the analogs (Figure 2A). These analogs have been tested *in vitro* using two different GBM cell lines (U87-MG and LN229). After 24-h treatment with these new analogs, cell viability was quantified as compared to MJ. First, different doses (100 μM, 500 μM, 1, 2, 5, and 10 mM) were applied for optimum dose determination. Proportionally, an equal volume of DMSO was used in the control group treatments. The analog C-10 caused the most effective cell death as compared to MJ treatments at the same concentrations. In the U87-MG cell line treated with 5 mM C-10, the cells showed 11% cell death while 61% of the cells were alive in the MJ-treated group of cells (Figure 3A). C-10 has an IC_50_ value of 3.2 mM for the U87 cell line (Figure 3B). Similarly, upon treatment with 5mM C-10, LN229 cells showed greater cell death (19%) than MJ treatments (67%) (Figure 3C). The IC_50_ value of MJ was 5.2 mM for the U87 cell line and 5.5 mM for the LN229 cell line. Next, the IC_50_ value of 3.2 was determined for the LN229 cell line (Figure 3D). These results showed that among 10 different analogs, 5 mM C-10 was the most active compound and more potential than MJ.

### 3.3 *C-10–*Treated GBM Cells Display Less Apoptotic More Necrotic Cell Populations

**FIGURE 4:**
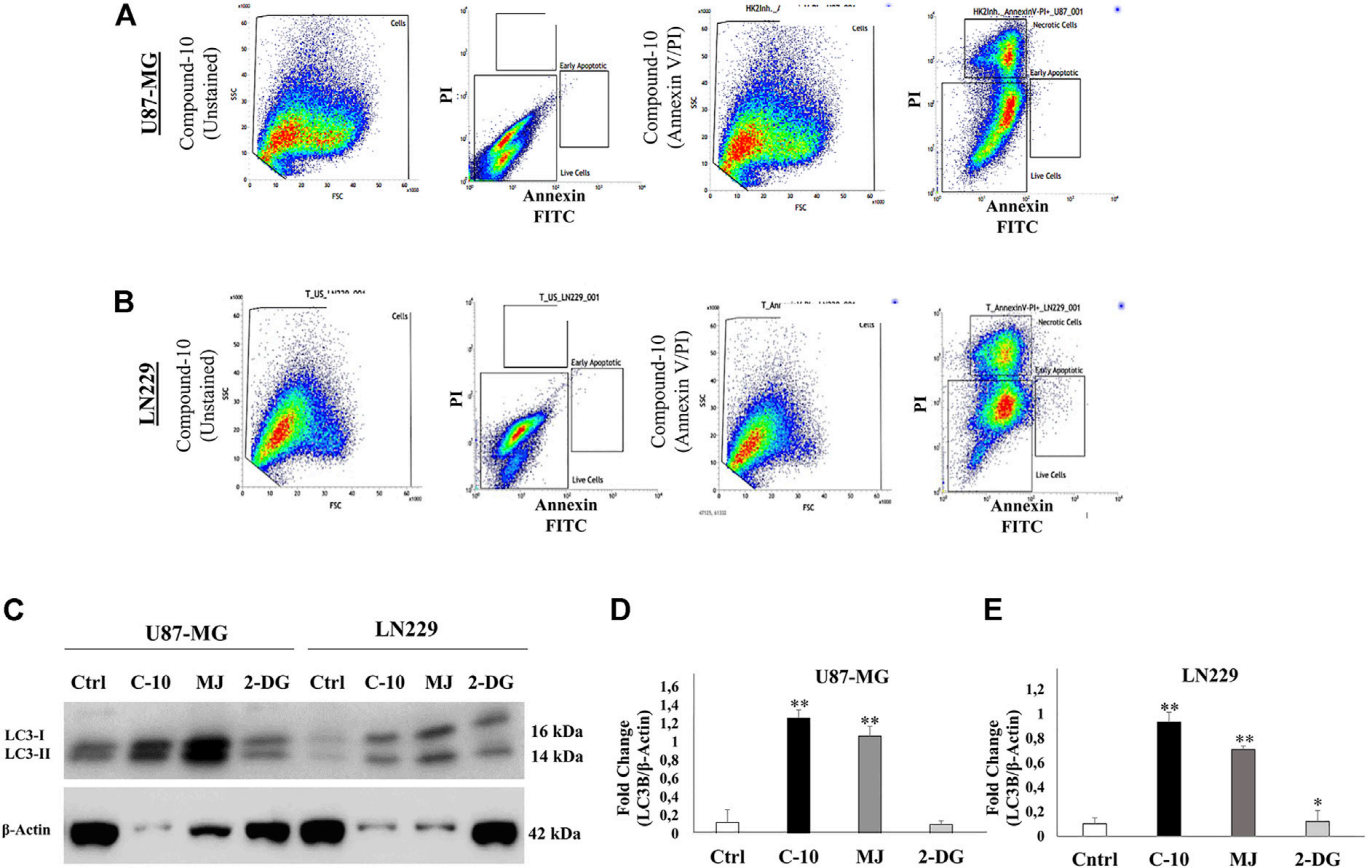
Compound-10 (C-10)–mediated cell death analysis in GBM cells. Flow cytometry analysis of cell death in U87-MG and LN229 cells after 24 h of treatment with C-10 (5 mM dose). Annexin-V or propidium iodide staining indicates apoptotic and necrotic cell death, respectively **(A,B)**. Detection of LC3I and LC3II, an autophagy marker, in GBM cells treated with C-10 by using the Western blot method **(C)**. Plot shows quantification of LC3I and LC3II levels by ImageJ (LC3I and LC3II band intensities are normalized to *β*-actin) in U-87 **(D)** and LN229 cells **(E)**.

According to cell viability results, we found that C-10 caused cell death in all GBM cell lines tested, U87 and LN229, more potently to known HK-II inhibitors, MJ and 2DG. Then, we further analyzed the cell death of GBM cells triggered by C-10. First, Annexin-V and PI staining was performed to determine the apoptotic and necrotic cell populations. When using Annexin-V for determining early apoptosis processes, PI was used to mark all dead cells. These two dyes were used together to exclude necrotic cells. Percentages of necrotic, early apoptotic, and apoptotic cells in both U87-MG cells (Figure 4A) and LN229 (Figure 4C) cells treated with 5 mM C-10 were determined by flow cytometry analysis. Accordingly, 2.03% of apoptotic cells and 33% of necrotic cells in U87-MG cells were counted (Figure 4B), while the proportion of apoptotic and necrotic cells in LN229 cells were 1.26% and 30%, respectively. (Figure 4D). These results indicated the presence of an undetectable cell population by Annexin-V and suggested that both cells should be evaluated for positivity of other cell death mechanisms. MJ was previously reported for its inducer role for autophagy (Zhang et al., 2016; Yin et al., 2020). Therefore, we further focused on 24-h C-10 treatment–based enrichment of autophagy in GBM cells.

### 3.4 Treatment With *C-10* Induces Autophagy and Enhances Death Signals in Both U87 and LN229 GBM Cell Lines

**FIGURE 5:**
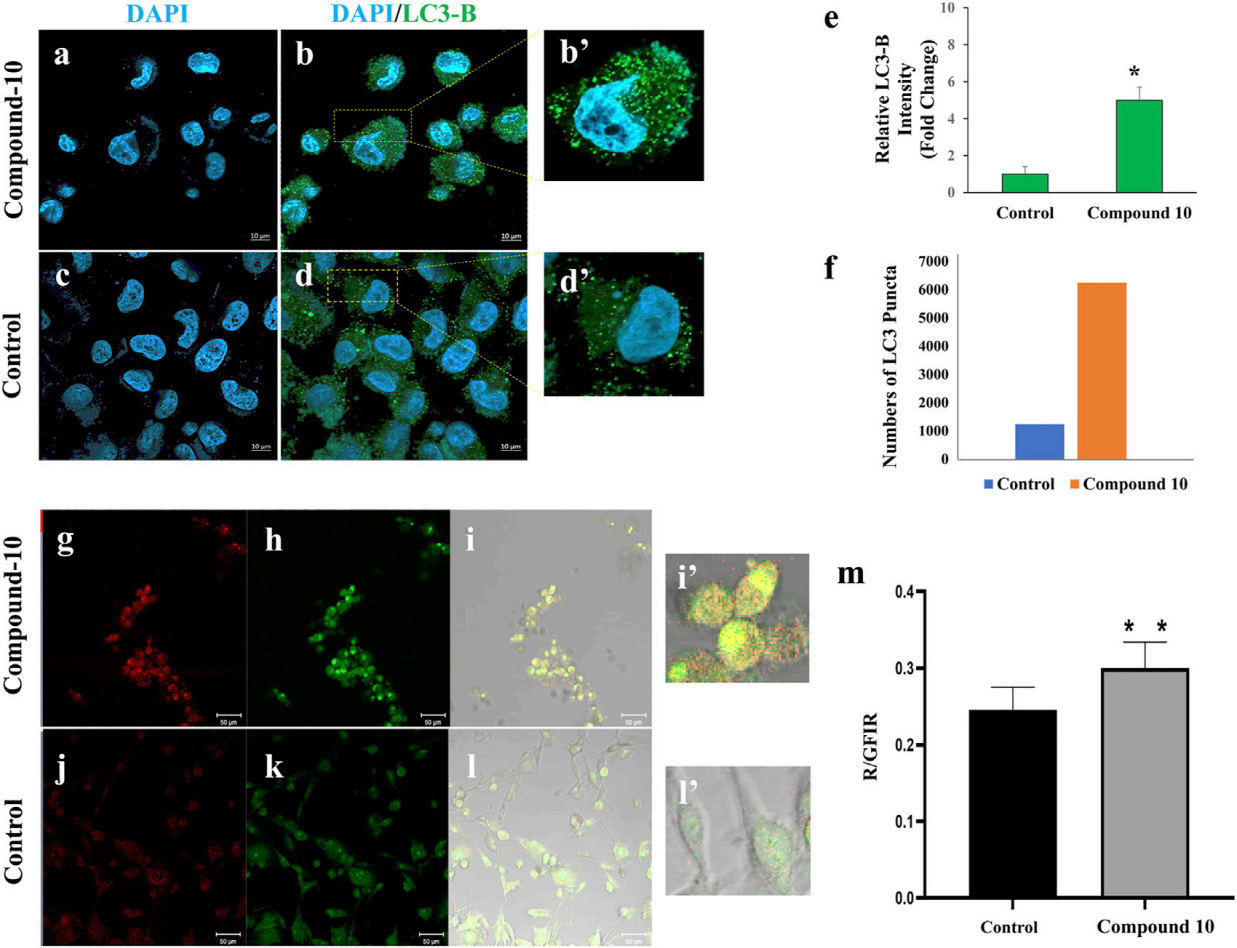
Autophagy detection by staining LC3 and acidic compartments in C-10–treated GBM cells. Immunofluorescence staining of LC3B (known as autophagy marker) in LN229 cells treated with C-10 **(A,B)** and no treatment **(C,D)** for 24 h. Plot showing the relative increase of LC3B compared to the control group **(E)**. Numbers of LC3B puncta were increased fivefold after C-10 treatment **(F)**. U87-MG cells were treated with C-10 for 24 h (only C-10 solvent was used in the control group). Acridine orange staining was used to detect autophagic cell death of the C-10–treated cells **(G-I)** and the untreated controls **(J-I)**. Ratiometric analysis (R/GFIR) was performed. Plot showing the rate of autophagic cell death compared to the control group **(M)**. [**(B’, D’, H’, and K’)** are three fold relative magnification].

In accordance with the results obtained in Annexin V–PI staining and Western blotting of LC3B, in another experimental set up, we further investigated whether C-10 caused autophagic cell death in the GBM cell lines. For this, C-10 treated LN229 cells and the control group were immunostained for LC3B protein, which is known to be in stringent association with the autophagosomal membranes formed in the autophagic process (Runwal et al., 2019). (Figures 5A–D,B’,D’). LN229 cells treated with the C-10 analog showed a 5-fold increase in LC3B dot intensity and numbers of LCB puncta compared to the control group (Figures 5E,F), (p*<0.01). Furthermore, in another GBM cell line, U87, we imaged autophagy by acridine orange staining. Conversion to orange indicates acidic compartments and was known as an indicator of autophagy (Figures 5G–L,I’, L’). Here, we showed that autophagy was detected in C-10–treated U87 cells and was significantly higher than that in untreated cells (Figure 5M), (p**<0.0001). Taken together, the novel MJ analog C-10 treatment led to increased autophagy in GBM cells.

## 4 Discussion

Hexokinase-II (HK-II), one of the hexokinases that have a major part in glycolysis, is overexpressed in many types of cancers (Pastorino and Hoek, 2008). A high expression of HK-II contributes to drug resistance in tumor cells, and the downregulation of HK-II develops chemosensitivity. This leads to metabolically overactive cancer cells and then promotes tumor growth and invasion. Therefore, treatment options targeting HK-II to suppress glycolysis have been a rising phenomenon in metabolism-based approaches against cancers. In the most malignant brain tumor, glioblastoma (GBM), which is refractory to current treatment modalities and is highly lethal, HK-II is overexpressed. For this aim, several HK-II inhibitors were designed and have been shown capable of reducing GBM tumor growth *in vivo*. In this study, we first examined the clinical significance of targeting HK-II in GBM patients by using glioma cohort data. HK-II high expressors showed decreased survival rates, and then we determined differentially upregulated genes and pathways. Next, in GBM cells, we analyzed the *in vitro* therapeutic efficacy and related cell death mechanism of our previously designed and synthesized MJ derivatives as HK-II inhibitors. In conclusion, our results showed that the novel HK-II inhibitor, compound 10 (C-10), functionally induces cell death moieties in GBM cells including autophagy, necrosis, and apoptosis and is more functional than MJ.

To determine the clinical significance of targeting HK-II in GBM cells, we examined patient data through TCGA. A bioinformatics analysis of glioma cohort data showed that HK-II overexpression in gliomas was prognostic with the up-regulation of already known associated markers with tumor progression/recurrence (such as VEGF, CXCL8, and HIF1A). The HK-II inhibitors may have an alternative/adjuvant effect to control tumor mass or recurrence in HK-II high-expressor GBM patients as the drugs in clinical use acting through DNA damage. This fact reveals the need for testing both available HK-II inhibitors and the development of more potent novel candidates to target HK-II activity in GBM cells. Extensive HK-II overexpression in GBMs provides a targeting strategy for drug designers, and to date, several drugs were synthesized and analyzed; of these, one widely used HK-II inhibitor is 2-deoxy-Dglucose (2-DG). Principally, 2-DG treatment triggers glucose accumulation and cell cycle arrest. Depending on the defined mechanism of action, 2-DG has been tested in many types of cancers, including GBM (Maschek et al., 2004). However, signs of toxicity have been observed in the brain (Singh et al., 2005). One of the most promising actors targeting HK-II activity is MJ and its derivatives. Principally, reactive oxygen species (ROS), MAPK signaling pathway, VDAC proteins, NF-κB pathway, and 5-LOX pathway are the main targets of MJ treatment in cancer cells (Cesari et al., 2014). MJ also affects the glycolytic pathway. To date, several MJ-derived compounds were reported with successful preclinical outcomes and some are in clinical trials. There is no acute toxicity effect of this drug reported in studies with humans and animal models (Cesari et al., 2014).

GBM cells are highly heterogenic for their ability of response to standard therapies, and they behave individually at the molecular level. In a previous study, we focused on the derivation of MJ derivatives as potential anticancer drug candidates. Our results showed that the novel HK-II inhibitors can target HK-II by blocking the interaction with VDAC on mitochondria and thereby, causing mitochondria toxicity and executing cell death in different cancer cells. Accordingly, in this study, we investigated the *in vitro* therapeutic effects of those 10 different MJ derivatives synthesized as compared to commercial HK-II inhibitors (MJ and 2-DG) on GBM cells. Among these 10 different molecules, the C-10 especially has been found to be the most potent MJ derivative in reducing cell viability compared to common HK-II inhibitors (MJ and 2-DG). The results were consistent with our previous data in different cancer cells (Sucu et al., 2019). Compared to MJ, the novel molecule C-10 was found more potent to reduce cancer cell viability. Then, we further analyzed its activity pattern in GBM cells considering its involvement of cell death moieties. The novel molecule C-10 showed the presence of both necrotic/apoptotic cell populations in GBM cells upon treatment with C-10 for 24 h. The viability plots indicated significantly induced cell death, while necrotic/apoptotic cell populations were distributed in different proportions within 24 h. In addition to this, autophagic cell death was reported previously for other MJ-derived compounds. Next, we designed a set of experiments to identify the possibility of an autophagy-related death mechanism of the cells in the dead population when exposed to C-10 for 24 h. Then, we showed the increased expression of LC3I (known as an autophagy marker) and LC3II (formed by cleavage of LC3I) in C-10–treated U87-MG and LN229 cells. Immunofluorescence imaging of LC3B and AO staining (which is frequently used in the detection of autophagy-induced cell death *via* monitoring acidic compartments) revealed that autophagy was upgraded by C-10 treatment. This may result in the increased dead cell population which could not be recognized by flow cytometry in 24 h, while cell death analysis revealed more dead cells in plots. These results demonstrate that in addition to apoptosis, autophagic cell death may also be induced within 24 h and conversely, apoptosis may be slightly involved in the beginning of death action and some proportion of the cells end up with necroptosis. In other words, increased apoptosis most likely can be seen in the later hours of the treatment, while in the 24-h death cell population is a result of activation of autophagy and necrosis. Potentially, the molecule can trigger cell death through apoptosis, necroptosis, and autophagy at different time points. A more detailed analysis of HK-2 inhibition and/or mitochondrial dysfunction–mediated cell death can reveal a well-defined mechanism of action. One of the possibilities is necroptosis which is a regulated necrotic cell death modality in a caspase-independent fashion and is mainly mediated by Receptor-Interacting Protein 1 (RIP1), RIP3, and Mixed Lineage Kinase Domain-Like (MLKL). Second, as the cells are in between apotosis–necrosis–autophagy, which is most likely oncosis linked to ATP reduction (as the cells need energy to be programed for apoptosis, in the absence of ATP they cannot enter into late apotosis). In oncosis, the cells cannot continue to caspase-mediated cell death; instead, they go through autophagy and end up with necrosis. Mechanistically, C-10 is an HK-2 inhibitor that eventually decreases glycolysis and acts on mitochondria, which may lead to decreased ATP and therefore possibly oncosis. Further examination of each scenario by sampling at different time points of treatments and analyses of downstream signaling events (to reveal the shifting of early apoptosis to the late stage of apoptosis or necrosis and analyze the non-apoptotic death fate of the cells) was carried out.

Moreover, targeting the cancer cell population which escapes from standard TMZ chemotherapy (TMZ functions as a DNA alkylating agent with no selectivity to cancer cells) using novel drug candidates is of high interest. Therefore, the construction of new molecules as more functional MJ derivatives and their clinically relevant working concentrations may provide combination therapy options since they have the ability to trigger different cell death mechanisms. A further examination of these derivatives with the combination of TMZ may also provide their sensitizing effects to the standard treatments in GBM cells.

In conclusion, the novel MJ derivative we previously developed, C-10, targets HK-II, and can trigger distinct cell death mechanisms in GBM cells, thereby providing greater cell death *in vitro* than MJ. Targeting glycolysis and downstream reactions by these MJ derivative HK-II inhibitors can potentially lead to a superior cell killing effect *via* including different cell death mechanisms in highly heterogenic and drug-resistant tumors such as GBM. Our primary results suggest that targeting HK-II overexpression in GBM patients by highly active MJ-derived compounds which can function at clinical doses may provide an option to standard therapy.

## Data Availability

The original contributions presented in the study are included in the article/Supplementary Material, further inquiries can be directed to the corresponding authors.
