# Supplementary figures and images for: Potential of Novel Methyl Jasmonate Analogs as Anticancer Agents to Metabolically Target HK-2 Activity in Glioblastoma Cells
Source: Front Pharmacol. 2022 May 23;13:828400. doi: 10.3389/fphar.2022.828400 (PMC9168889; doi:10.3389/fphar.2022.828400)

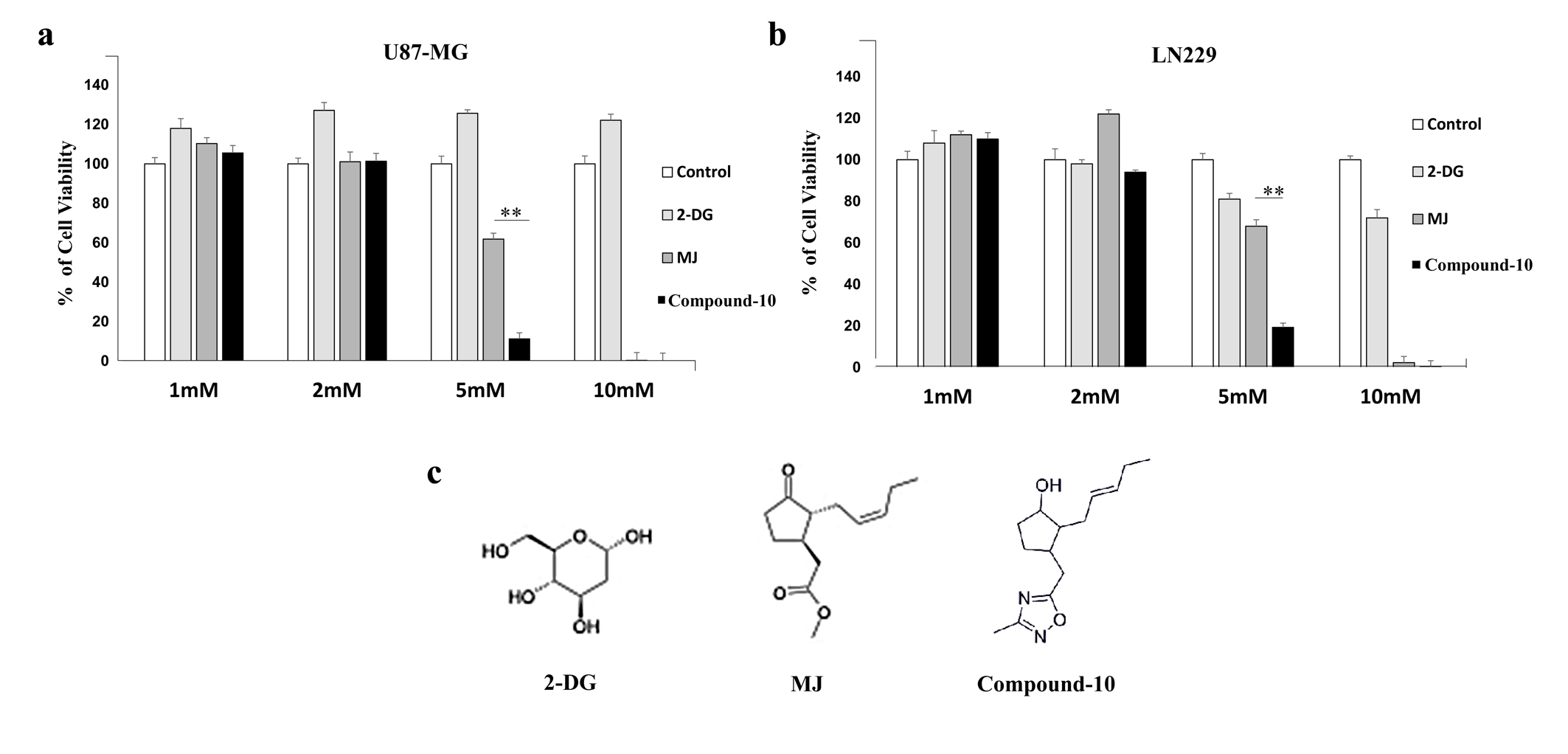

Supplement: Supplementary file 1 [file Image1.tif]
